# Aminoaciduria in the prediction of ifosfamide-induced tubulopathy after childhood cancer: a feasibility study

**DOI:** 10.1186/s40814-015-0040-0

**Published:** 2016-01-22

**Authors:** Jessica E. Morgan, Karl McKeever, Kay S. Tyerman, Michael Henderson, Susan Picton, Robert S. Phillips

**Affiliations:** 1 Department of Paediatric Oncology, Leeds Teaching Hospitals NHS Trust, Leeds, UK; 2 Centre for Reviews and Dissemination, University of York, Leeds, UK; 3 Department of Paediatric Nephrology, Royal Belfast Hospital for Sick Children, Leeds, UK; 4 Department of Paediatric Nephrology, Leeds Teaching Hospitals NHS Trust, Leeds, UK; 5 Department of Biochemical Genetics, Leeds Teaching Hospitals NHS Trust, Leeds, UK

**Keywords:** Ifosfamide, Oncology, Renal damage, Long-term follow-up

## Abstract

**Background:**

Ifosfamide, an alkylating agent used widely in the treatment of childhood malignancy, can cause many side effects including a proximal tubulopathy. Studies suggest that aminoaciduria is seen most commonly of all the biochemical abnormalities of ifosfamide-induced tubulopathy. A recent systematic review has found a paucity of data regarding the value of early markers indicating clinically significant tubulopathy. We undertook a pilot study to determine the feasibility of examining whether patients can be risk-stratified on the basis of aminoaciduria for the development of future significant ifosfamide-induced tubulopathy, to allow the evolution of appropriate follow-up strategies. We also aimed to define accrual rates, costs and clinical demands for a future larger study.

**Methods:**

This observational study recruited 21 patients from the Leeds Paediatric Oncology service. The medical notes of each patient were reviewed for demographic and clinical data. Simultaneous samples of blood and urine were obtained.

**Results:**

The investigations in the feasibility study were acceptable to patients and were minimally demanding on both clinical and laboratory staff. Financially, the cost per patient was minimal. This study was not powered to detect significant associations with TmP/GFR (ratio of renal tubular maximum reabsorption rate of phosphate to glomerular filtration rate), growth and electrolyte supplementation. However, all patients with minimal aminoaciduria (≤2 elevated urinary amino acids) had normal TmP/GFR and no need for electrolyte supplementation.

**Conclusions:**

This pilot study has shown that a larger study is feasible and may provide clinically useful data to change current practice. This should aim to establish whether the number of abnormal amino acids or the degree of abnormality is most significant in predicting clinically significant proximal tubulopathy.

## Background

Ifosfamide is an alkylating agent that is used widely for the treatment of malignancies, including those seen in children. Ifosfamide can cause many immediate and some long-lasting side effects, including a proximal tubulopathy and renal impairment [1]. The proximal tubulopathy results in phosphaturia, glycosuria, aminoaciduria, hypophosphataemia, hypokalaemia and a hyperchloraemic metabolic acidosis [2]. Clinically, there is a spectrum of proximal renal tubular disease ranging from clinically insignificant glycosuria and mild phosphaturia to a more severe generalised tubulopathy which is clinically significant and requires increased fluid intake, along with potassium, phosphate and bicarbonate supplementation [3]. If unrecognised, severe tubulopathy leads to poor growth, general malaise and metabolic bone disease which can result in pathological fractures [4, 5]. The majority of patients will develop a temporary acute proximal tubulopathy whilst receiving ifosfamide. However, the prevalence of long-term ifosfamide-induced tubulopathy has not yet been clearly defined, with estimates of 6–46 % of patients who have received ifosfamide as treatment for cancer [6].

Current long-term follow-up programmes aimed to detect renal damage in children who have received ifosfamide vary. Studies suggest that aminoaciduria is the most frequently detected abnormality in tubular dysfunction and hence may be a good early predictor of long-term ifosfamide-induced tubulopathy [7]. The presence of aminoaciduria is due to an impairment of the mechanisms which would normally result in the reabsorption of amino acids from the filtrate in the proximal tubule [3].

A recent systematic review performed by this group has looked at the accuracy and utility of early markers of ifosfamide-induced proximal tubulopathy [6]. This review found a general paucity of data in this area. The evidence that is available suggests that low levels of aminoaciduria indicate a low risk of developing a clinically significant tubulopathy. This review also found that urinary β2 macroglobulin might be useful in a similar way, with low levels suggesting a low risk of significant tubulopathy and high levels identifying patients at higher risk who require more intense follow-up. However, the evidence for the use of urinary β2 microglobulin is even scarcer than that for aminoaciduria.

We undertook a pilot study to determine the feasibility of performing a larger more definitive study into this question. Specifically, we aimed to determine the patient accrual rate, to monitor data quality of samples when processing the results in the laboratory, to determine the costs associated with these extra investigations and to assess the extra demand on clinical and laboratory staff. Finally, we hoped to determine whether there is an indication that generalised aminoaciduria is a good negative predictive marker for long-term tubulopathy so as to justify the larger future study.

The final study would aim to establish whether patients can be risk-stratified for the likelihood of future significant ifosfamide-induced tubulopathy. This would then allow more tailored follow-up strategies for patients deemed to be at risk of developing a clinically significant tubulopathy following completion of chemotherapy. This may improve the speed of detection of at-risk patients whilst simultaneously reducing the burden of investigation for those patients who are at low risk of developing this complication of their treatment.

## Methods

This study was assessed and approved by an NHS Research Ethics Committee (NHS REC Leeds West Ref 10/H1307/22) prior to commencement. Leeds Teaching Hospitals NHS Trust is a tertiary referral hospital and provides care for approximately 110 new children and young people diagnosed with malignancy per year. All patients within the Regional Oncology Service in Leeds who had completed ifosfamide chemotherapy between 2003 and 2012, and were between 3 months and 5 years from completing treatment, were identified using local records. This was considered to be adequate to assess the feasibility within the clinical environment and the laboratory along with the potential uptake rate by patients. These children had been treated according to standard national chemotherapy protocols for their disease. The only exclusion criterion was the presence of renal impairment or tubulopathy prior to the commencement of treatment with ifosfamide. Information leaflets were sent to identified patients through the post, and patients were then approached at their next clinic appointment to determine their decision regarding participation. All included patients provided written informed consent to participate.

After obtaining consent, the medical notes of each patient were reviewed for the demographic and clinical data included in Table 1. Simultaneous samples of blood and urine were obtained, and investigations as per Table 2 were performed during their next, routinely planned, clinic appointment. Height and weight were measured using calibrated equipment within the clinic, by staff regularly trained in the taking of these measurements. Growth velocity was measured for the year before study enrollment and was compared to Tanner and Davies charts [8].

**Table 1:** Demographic and clinical data collected

Age (years), sex and ethnicity of patient	
Date of data collection	
Underlying diagnosis	
Cumulative dose of ifosfamide received (g/m^2^)	
Other chemotherapeutic agents used	
Time elapsed since completion of ifosfamide and other chemotherapeutics (months)	
Current medications	
Complications (poor growth/fractures)	
Growth velocity in year prior to investigations (cm/year)

**Table 2:** Investigations performed

Urea and electrolytes, serum creatinine, calcium, phosphate, magnesium	
Urinary osmolality	
Urinary amino acids	
Urinalysis for proteinuria and glycosuria	
Ratio of renal tubular maximum reabsorption rate of phosphate to glomerular filtration rate (TMP/GFR)	

For each patient, the degree of aminoaciduria was classified as normal (≤2 amino acids above the normal range), mild (3–7 amino acids above the normal range) or generalised (≥8 amino acids above the normal range) [7]. Any patients who were detected to have significant aminoaciduria and/or abnormal serum electrolytes or bicarbonate were contacted and commenced on supplementation under the direction of the paediatric nephrology team. The definition of proximal tubulopathy was a reduced TmP/GFR (ratio of renal tubular maximum reabsorption rate of phosphate to glomerular filtration rate) below the normal range for age, reduced growth velocity ≤3rd centile for age or need for electrolyte supplementation.

### Statistical analysis

Basic descriptive analysis was performed on the demographic data of the patients. The degree of aminoaciduria was analysed against electrolyte supplementation, growth velocity ≤3^rd^ centile for age and TmP/GFR less than the normal range using the chi-square test. Spearson’s rank correlation coefficient was calculated for the relationship between number of raised amino acids and TmP/GFR. Analysis was performed using SPSS software (IBM, version 22, Armonk, NY). A *p* value of 0.05 was defined as the threshold for significance, and 95 % confidence intervals are provided where appropriate. It is recognised that these results are preliminary given the small sample size.

In order to determine the feasibility of a larger study, we also collected data on the costs for each patient, the steps involved in processing results and the practical challenges encountered through the performance of this pilot study. The demands on clinical and lab staff were assessed through ongoing discussion with the involved parties. In particular, the laboratory staff time involved in the interpretation of chromatography was assessed so as to establish the demands for this part of the service.

## Results

Information leaflets were sent to 39 patients, of these, 24 consented to be included in the study. One patient consented to enter the study but did not complete the investigations required. Two patients were excluded as their samples could not be processed for technical reasons. Full study data was available for 21 patients.

### Feasibility of the full study

Overall accrual to the study was 21 of 39 (54 %) of invited patients. Originally, we had intended to measure urinary β2 microglobulin, but the requirement of special preparation of patients to ensure alkaline urine was considered to be inappropriate for routine use and therefore was not performed.

**Fig. 1:**
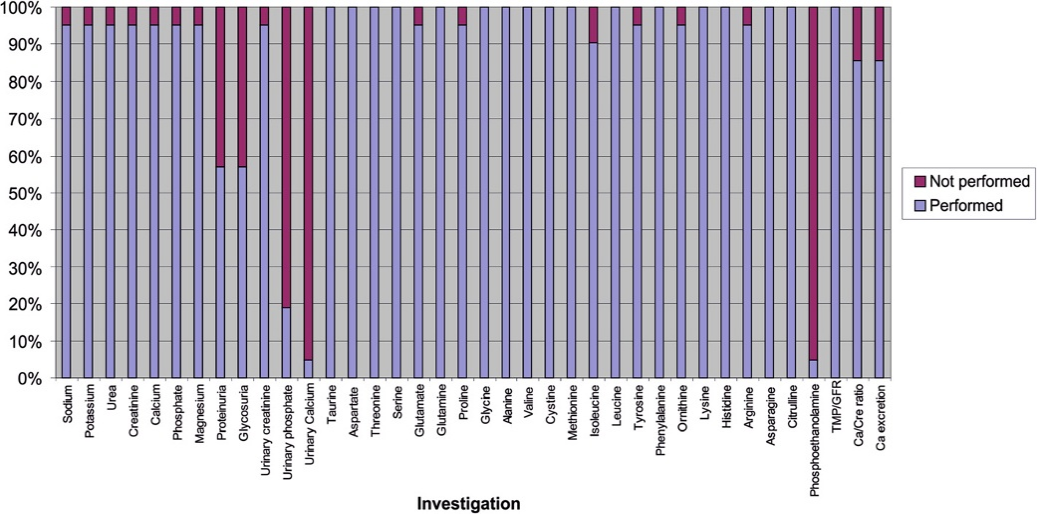
Investigations performed

The data collected was generally of good quality with fewer than 10 % of samples unable to be processed. Notably, the most poorly recorded item of data was the urine dipstick (only available for 12 of 21 patients); it is unclear whether this was not performed or simply not documented (see Fig. 1)

Reports from laboratory staff are that there was minimal disruption to the usual service as the number of patients involved was small. Each sample requires the following steps to be taken. Firstly, sample preparation including precipitation, spinning, loading and placing onto analyser, preparing the analyser and inputting samples to software must take place. On average, this takes 30 min for six samples. Then the sample is run through the analyser. This takes 3 h. Next, the quantitative results must be evaluated and verified. A senior member of staff familiar with chromatography has to review all the peaks in each chromatogram and ensure that the quantification parameters are correct. Finally, the data must then be manually entered into the reporting system. These final processes take 20 to 30 min per sample.

There were some problems within the laboratory because TmP/GFR is performed so infrequently that the staff booking the samples into the laboratory failed to recognise what tests were required for 6 of the 21 samples. This resulted in laboratory staff having to spend extra time chasing up results so that the TmP/GFR could be derived [5].

The cost of the study investigations was £35.75 per patient. Administration costs were included within the usual departmental budget. They amounted to less than £100 for all patients.

### Clinical results

**Fig. 2:**
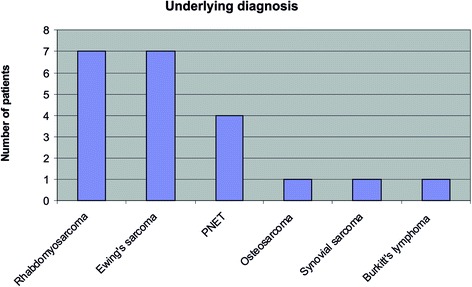
Underlying diagnoses

The demographic characteristics of the study patients are detailed in Table 3. The range of underlying diagnoses is seen in Fig. 2.

**Table 3:** Demographic characteristics of patients

Male: female	10:11
Median age	10.9 years (range 4–19 years)
Ethnicity	19 white British 1 Indian 1 ‘any other mixed background’
Mean cumulative dose of ifosfamide (g/m^2^)	61 (range 16.5–102)
Time between last dose of ifosfamide and sample collection (months)	Median 25 (range 3–107)

Of the 21 patients included in the study, 7 (33 %) had normal, 5 (24 %) had mild and 9 (43 %) had generalised aminoaciduria. The degree of aminoaciduria did not show statistically significant association with the need for electrolyte supplementation (*p* = 0.096), growth velocity ≤3rd centile for age (*p* = 0.92) or TmP/GFR less than the normal range (*p* = 0.24).

No patients with no or mild aminoaciduria required electrolyte supplementation (95 % confidence interval (CI) 0–35 % for no aminoaciduria, 95 % CI 0–43 % for mild aminoaciduria); 33 % of patients with generalised aminoaciduria required electrolyte supplementation (95 % CI for proportion 12–65 %). When growth velocity ≤3^rd^ centile for age was assessed, 29 % (95 % CI 8–64 %) of children with no aminoaciduria, 40 % (95 % CI 12–77 %) of children with mild aminoaciduria and 33 % (95 % CI 12–65 %) of children with generalised aminoaciduria had poor growth. No patients (95 % CI 0–35 %) with no aminoaciduria had low TmP/GFR; 20 % (95 % CI 4–62 %) of children with mild aminoaciduria and 33 % (95 % CI 12–65 %) of children with generalised aminoaciduria had low TmP/GFR.

However, when examining the raw data looking at the need for electrolyte supplement and TmP/GFR, the trend suggests that aminoaciduria does indicate abnormalities in these areas which this study is not powered to detect. Indeed, only one patient of the four patients with decreased TmP/GFR did not have generalised aminoaciduria, having only six raised urinary amino acids but did have three other amino acids excreted at the upper limit of the normal range. He also had proteinuria and glycosuria on urine dipstick but did not require electrolyte supplementation. One patient with low TmP/GFR required electrolyte supplementation with phosphate and calcium.

Notably, patients with minimal aminoaciduria (≤2 amino acids above the normal range) had no need for electrolyte supplementation and normal TmP/GFR.

**Fig. 3:**
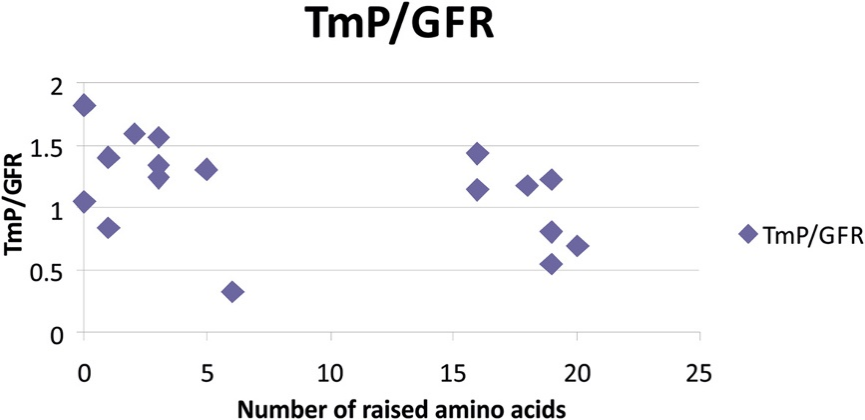
Correlation between aminoaciduria and TmP/GFR

The scatter graph in Fig. 3 demonstrates the correlation between aminoaciduria and TmP/GFR. The Spearman’s rank correlation coefficient for this relationship is −0.396 (*p* = 0.075), which suggests that an increased number of abnormal amino acids may be associated with lower TmP/GFR.

Six children had received cyclophosphamide within their chemotherapeutic regimes. Two of these had generalised aminoaciduria, of which one had low TmP/GFR and low growth velocity, two had mild aminoaciduria, of which one had low TmP/GFR and two had normal levels of amino acid excretion, of which one had low growth velocity. None of these children required electrolyte supplementation.

Three children received platinum chemotherapy. One, who had also received cyclophosphamide, had generalised aminoaciduria, low TmP/GFR and low growth velocity. One had mild aminoaciduria, normal TmP/GFR and low growth velocity. The final child had normal amino acid excretion levels, normal TmP/GFR and normal growth velocity.

## Discussion

### Feasibility

This protocol was generally well accepted by patients as it required little extra investigation, with only one additional blood and urine test beyond current surveillance. The study was also minimally demanding on both clinical and laboratory staff. Financially, the cost per patient was minimal. The omissions in data could be reduced by creating structured follow-up schedules which staff could easily follow within the clinic. Over time, this would become part of standard procedures and therefore be less likely to be forgotten or undocumented.

### Clinical results

The data from these patients showed no significant correlation between aminoaciduria and need for electrolyte supplementation, growth velocity ≤3^rd^ centile for age or TmP/GFR less than the normal range. However, the raw data does suggest that generalised aminoaciduria trends towards a need for electrolyte supplementation and also a lower TmP/GFR. The finding that patients with minimal aminoaciduria had no need for electrolyte supplementation and had normal TmP/GFR needs to be confirmed, as this may have significant clinical applications.

### Comments on limitations and generalisability of the study

This study was limited by the number of patients involved, which was a result of being run in a single centre. This reduced the power of the statistical calculations such that significance was not met. The larger study from this pilot should be designed to overcome this problem. Furthermore, this study had a majority of White British patients and therefore may not be generalizable to those from Black and ethnic minority groups. The main study should aim to increase the number of patients recruited from this population.

Finally, although we have described the use of other nephrotoxic chemotherapeutic agents, given the small numbers of patients involved, no conclusions can be drawn from this. Consideration should be given to the role of these drugs and other nephrotoxic medications used in combination with ifosfamide within future studies.

The feasibility of this study has been established, with minimal clinical or laboratory issues. However, generalising this practice to other centres will need consideration of local systems and protocols. Establishing a multi-centre study will hold significant challenges that have not been explored by this pilot, including centralisation of data, data storage, selection of patients across centres and other costs. The future study should also aim to have firm criteria for the use of electrolyte supplementation so as to ensure consistent findings and therefore outcomes across all patients. Furthermore, the future study should aim to take into account the potential changes in tubulopathy over time and may aim to identify the most appropriate time point for screening. Previous works by Rossi et al. and Skinner may inform this work [7, 9].

When considered in light of the recent systematic review, the prevalence of generalised aminoaciduria within this sample was within the range previously reported [6]. In particular, we can compare this study with that done by Rossi et al., as measuring similar variables [7]. We found 43 % of patients to have generalised aminoaciduria compared with 28 % in Rossi’s sample. Meanwhile, 19 % of patients we studied had a TmP/GFR below the normal range, whilst Rossi et al. reported 17.3 % had severe impairment of phosphate reabsorption. The two studies are different, in that we measured only one episode of data for each patient, compared with multiple data points in the work of Rossi et al. We also had different periods of follow-up per patient and this may reflect a different phase in the development of proximal tubulopathy following ifosfamide. Rossi et al. also found that the absence of generalised aminoaciduria was not associated with significant tubulopathy.

Overall, the suggestion from this study is that the absence of generalised aminoaciduria is a good negative predictive marker for long-term tubulopathy.

## Conclusions

This pilot study has shown that the intended main study is feasible and may provide clinically useful data with potential to change current practice. A power calculation has been completed for the proposed main study. This calculation was based on a cohort of 100 patients and a 7 % prevalence of a clinically significant tubulopathy (proposed primary outcome measure for main study). With a sensitivity of 91 % and a specificity of 75 %, we estimate that a population of 180 patients will be needed to provide an adequately powered study. We estimate that this may take 3 years if performed as a multi-centred trial of ten centres with 12 new eligible patients seen per year per centre (with 54 % accrual). Future studies should aim to establish whether the number of abnormal amino acids or the degree of abnormality is most significant in predicting long-term clinically significant proximal tubulopathy.

Furthermore, other specialties may wish to investigate the use of aminoaciduria as a screening tool for other patients at risk of drug-induced tubulopathy, for example those treated with sodium valproate for seizures.

## Abbreviation

TmP/GFR: ratio of renal tubular maximum reabsorption rate of phosphate to glomerular filtration rate
